# Hemichorea in a patient with ipsilateral cortical infarction: a case report

**DOI:** 10.1186/s12883-021-02420-4

**Published:** 2021-10-29

**Authors:** Jie Wei, Yue Zhang

**Affiliations:** 1 Department of Neurology, 905th Hospital of PLA Navy, No 1328 Huashan Road, Changning District, Shanghai, 200052 China; 2 Department of Neurology, Huashan Hospital, Fudan University, No.12 Wulumuqi Road, Jing’an District, Shanghai, 200040 China

**Keywords:** Hemichorea, Hemiballismus, Ipsilateral, Stroke, Cortical infarction

## Abstract

**Background:**

Hemichorea is usually caused by contralateral deep structures of brain. It rarely results from acute cortical ischemic stroke and that caused by ipsilateral brain lesions is even rarer.

**Case presentation:**

A 64-year-old female presented with acute obtuseness and left-sided hemichorea. She had a history of right frontal lobe surgery and radiotherapy due to brain metastasis from lung cancer 8 years ago. MRI revealed acute left frontal lobe infarction in addition to an old right frontal lobe lesion. 18FDG PET-CT showed hypometabolism in the left frontal lobe and hypermetabolism in the right basal ganglia region and central sulcus. The choreatic movement remitted after antipsychotic treatment.

**Conclusion:**

The mechanism of hemichorea after ipsilateral cortical infarction is poorly understood. We assume both previous contralateral brain lesion and recent ipsilateral ischemic stroke contributed to the strange manifestation in this case.

**Supplementary Information:**

The online version contains supplementary material available at 10.1186/s12883-021-02420-4.

## Background

Hemichorea is an uncommon manifestation of acute ischemic stroke [1]. The responsible lesions usually involve deep structures of brain, such as subthalamic nucleus (STN) or striatum, but cortical infarction can also give rise to hemichorea or hemiballismus [1–4]. However, to the best of our knowledge, cases with ipsilateral hemichorea or hemiballismus after stroke are extremely rare [5–7]. We describe a peculiar case with ipsilateral hemichorea and hemiballismus after acute frontal cortical infarction.

## Case presentation

**Fig. 1:**
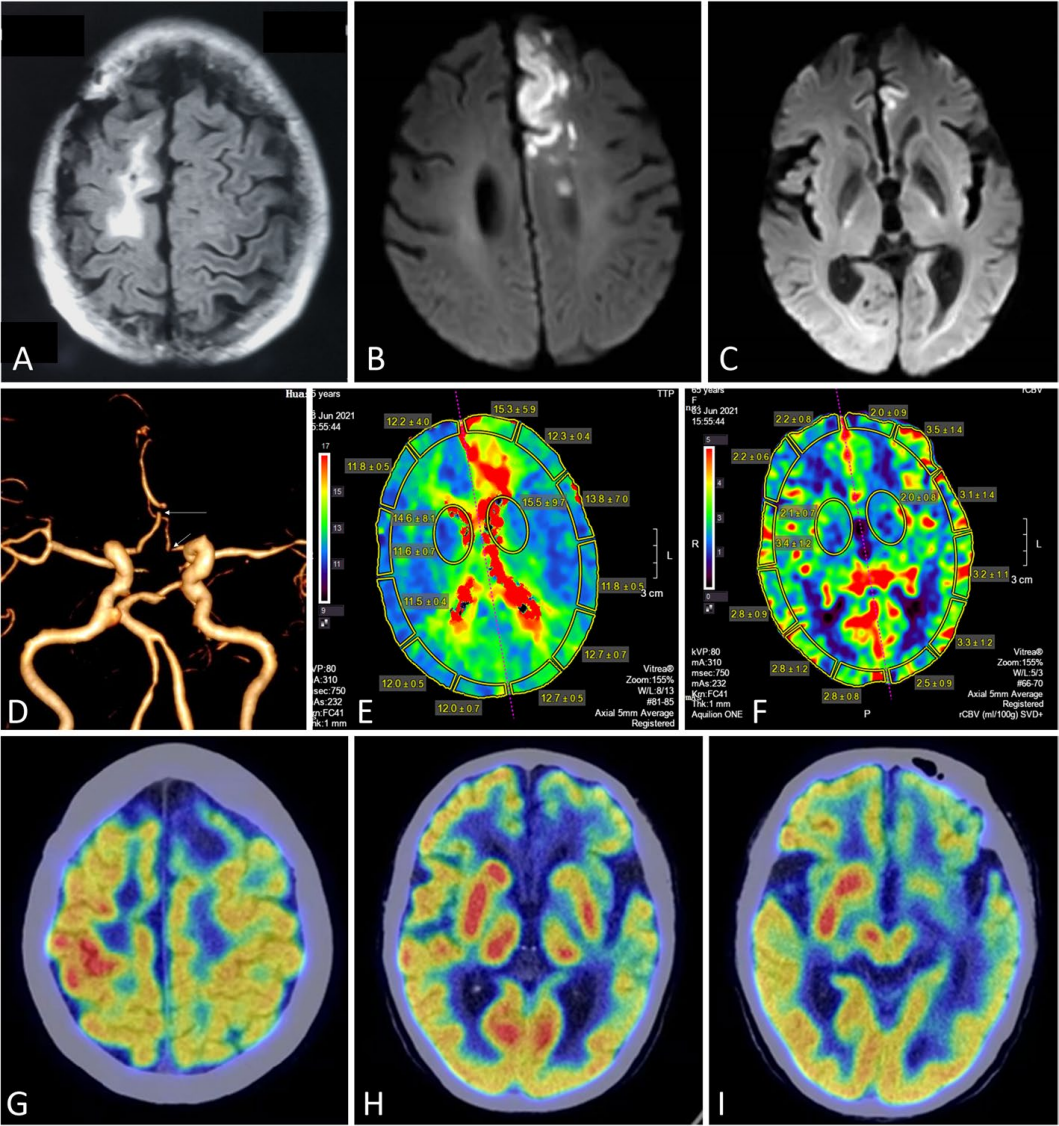
**A**. Fluid attenuated inversion recovery sequence showed an old lesion in the left frontal lobe 9 days before admission. **B** and **C**. Diffusion weighted imagines demonstrated acute left frontal lobe infarction without basal ganglia region involvement after admission. **D**. CT angiography revealed stenosis in both anterior cerebral arteries (arrows). **E** and **F**. CT perfusion revealed delayed perfusion in the left frontal cortex, as shown in the time-to-peak (TTP) with normal perfusion of deep structures. **G**, **H** and **I**. 18FDG PET-CT showed hypometabolism in the left frontal lobe and hypermetabolism in the right central sulcus and basal ganglia region

A 64-year-old female began to suffer from paroxysmal left arm twisting 2 months ago. The abnormal movement usually last less than 30 s and occurred once or twice a week. Brain MRI revealed nothing other than an old lesion in the right frontal lobe 9 days before admission (Fig. 1 A). She had no limbs weakness, numbness or sleep disorders. Five days ago, she developed obtuseness and sustained left-sided choreatic movements after she woke up in the morning. She was admitted on May 26, 2021. Past history was remarkable for brain metastasis from lung adenocarcinoma to the right frontal lobe in 2013. She received right frontal mass biopsy and radiotherapy in addition to systemic chemotherapy. However, detailed protocol of the radiotherapy was unavailable. Complete remission was achieved after treatment and she had no obvious neurological symptoms. The frontal lobe lesion on MRI remained unchanged during the past 8 years. None of her family members had similar problems.

On neurological examination, the patient was obtuse and uncooperative. No obvious ocular movement disturbance was observed. Pupils were qual, round, and reactive to Light. Bilateral nasolabial grooves were symmetric. Left-sided hemichorea was irregular, rapid, random, unpredictable and of high amplitude (Supplemental material. Video). Her face and tongue were not affected. No myoclonus or facial dystonia were observed. Right biceps reflex was 3+ and knee jerk was 2+. Muscle strength of right limbs was graded at least 4/5. Reflexes in her left extremities could not be evaluated due to hemichorea. Muscle strength of left limbs was graded at least 3/5. Babinski signs were present bilaterally. Sensory, cerebellar functions, and gait were unable to be evaluated. Laboratory tests which included blood glucose, liver function, renal function, thyroid hormones, parathyroid hormone, electrolytes, ammonia, ceruloplasmin, tumor markers (including CEA, NSE, CA125, CA199, etc.), anti-streptolysin-O antibody, anti-cardiolipin antibodies, anti-nucleus antibodies, anti dsDNA antibodies, antibodies to HIV and syphilis were negative or in normal range. Lumbar puncture revealed opening pressure of 220 mmH_2_O. Cerebrospinal fluid (CSF) was acellular with slightly elevated protein level. Malignant cells were not found in CSF. Commercially available paraneoplastic antibody panel (anti Hu, Yo, Ri, GAD65, Ma2, CV2, Amphiphysin, Tr, ANNA-3, PCA) and autoimmune encephalitis antibody panel (anti NMDAR, AMPA1, AMPA2, LGI1, CASPRA2, GABAb, DPPX, lgLON5, mGluR5, MOG) were negative. Lung CT showed no sign of tumor relapse. Paraneoplastic syndrome was initially suspected, but a repeat brain MRI revealed acute infarction in the left frontal lobe, indicating problem in the left anterior cerebral artery (Fig. 1B, C). No abnormalities were detected in the basal ganglia on T1-weighted images, CTA revealed 90% stenosis of both anterior cerebral arteries (Fig. 1D). CT perfusion revealed decreased cerebral blood flow (CBV) in the left frontoparietal lobe but not in the bilateral basal ganglia regions (Fig. 1E, F). Total body 18FDG PET-CT showed no sign of cancer relapse but hypometabolism in the left frontal lobe and hypermetabolism in the right basal ganglia region and central sulcus (Fig. 1G-I). Electroencephalogram demonstrated diffuse θ waves, prominent in the left side. The patient was diagnosed with post-stroke hemichorea-hemiballismus and was given risperidone 1 mg QD along with aspirin 100 mg QD and atorvastatin 20 mg QN. Hemichorea soon alleviated and disappeared 1 week later. Neurological examination found muscle strength was 5/5 in all extremities.

## Discussion

**Fig. 2:**
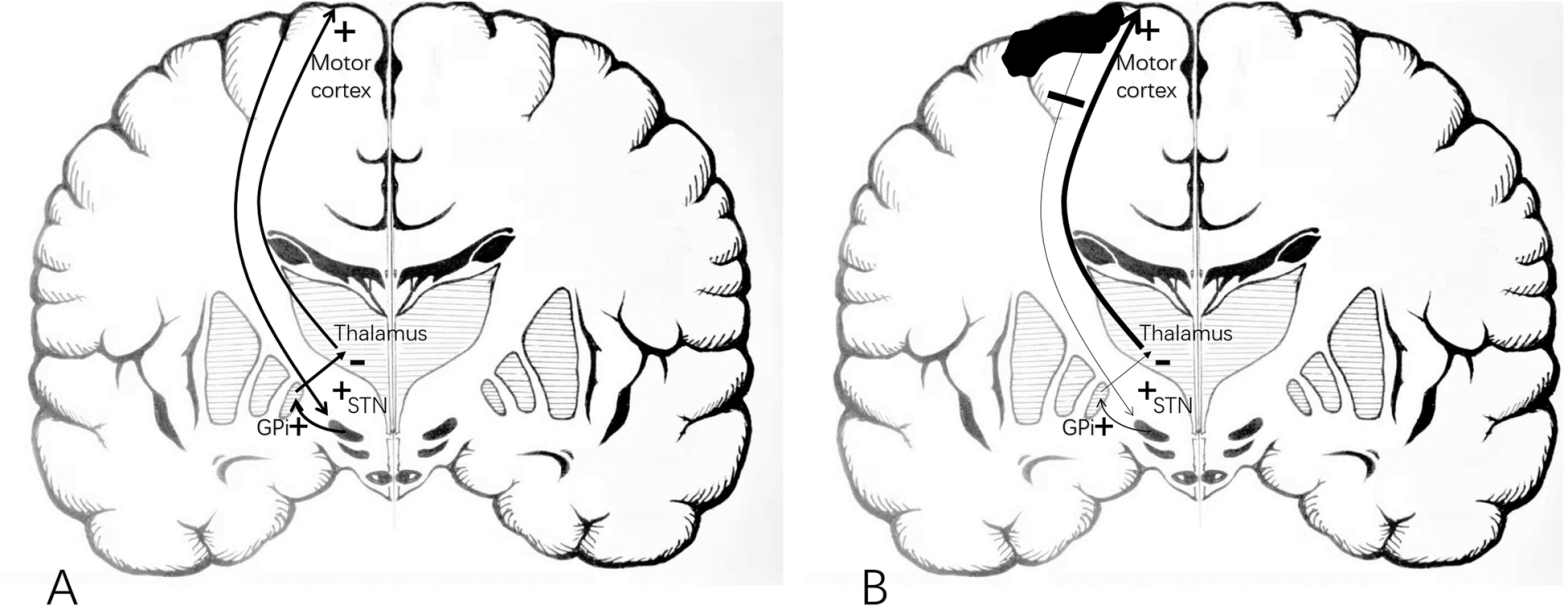
The subthalamic nucleus (excitatory) is directly activated by cortical inputs, enhancing the inhibitory activity of the globus pallidus internal. This leads to increased inhibition of the thalamus, and then decreased motor response. **B**. When the cortex is impaired, the inhibition of thalamus decreases, and then motor response increases. The picture was drawn by Mr. Wende Zhang

Hemichorea is a rare symptom of acute ischemic stroke. Chung SJ, et al. reported the incidence of post-stroke hemichorea was 0.54% [1]. In our case, sustained hemichorea was preceded by paroxysmal hemichorea. We assume this was possibly limb shaking TIA which takes the form of paroxysmal involuntary hyperkinetic movement. Limb-shaking TIA presenting as hemichorea-hemiballismus have been described [8]. Most post-stroke hemichorea is related to lesions in the basal ganglia region, especially the STN or lentiform nucleus. However, cases of hemichorea caused by cortical infarction have been occasionally reported [1–4]. The mechanism by which cortical lesions result in hemichorea is not well understood. One hypothesis is that hypoperfusion of the basal ganglia without evident lesions on MRI may be the underlying cause, but several studies proved cortical infarction alone was adequate to cause hemichorea [3, 9]. In our case, CT perfusion did not detect hypoperfusion in basal ganglia regions, either. The other hypothesis is disturbance in the hyperdirect way may cause hemichorea [3]. In this pathway, the STN (excitatory) is directly activated by cortical inputs, enhancing the inhibitory activity of the globus pallidus internal (Fig. 2A) [10]. So, decreased cortical signal output due to cortical infarction may induce choreatic movements (Fig. 2B).

Up to our knowledge, very few post-stroke cases with ipsilateral hemichorea have been reported in the literature and these cases are not impeccable. Two cases described by Borgohain R, et al. [6] and Kannepalli NR, et al. [7] presented with contralateral hemiplegia besides ipsilateral hemiballismus. Biballism was possible since contralateral hemiplegia may mask the contralateral ballistic movements [6, 7]. Radiological tools before appearance of MRI might miss small lesions in early cases [5, 6]. On the contrary, our patient had no contralateral hemiplegia and MRI can rule out small brain lesions, thus, we assume ipsilateral hemichorea is possible. Inatomi Y proposed 3 types of possible pathomechanisms which may explain ipsilateral hemiparesis: 1) absence of crossed corticospinal tracts (CST) (type I), 2) damage of the uncrossed CST (type II), 3) recent damage of the ipsilateral uncrossed CST with previous injury in the contralateral crossed CST (type IIa) [11]. In our case, a mechanism similar to type IIa injury is likely since the patient had a history of right frontal lobe biopsy and radiotherapy 8 years ago. Damage of the right crossed CST might activate left uncrossed CST. When the left hyperdirect pathway was impaired due to recent left frontal lobe infarction, disinhibition of left primary motor cortex gave rise to left-sided hemichorea through left uncrossed CST. Other possible mechanisms include lesions that affected the secondary motor area which bilaterally innervate the face and limbs [12], cortical reorganization within the motor areas of the unaffected hemisphere [13] or involvement of ipsilateral fibers with double decussation [12].

In the present case, 18FDG PET-CT showed hypometabolism in the left frontal lobe which obviously resulted from cortical infarction, while hypermetabolism in the right basal ganglia region and cortex might not reflect blood flow changes since CT perfusion was normal in these areas. Basal ganglia hypermetabolism has been found in patients with reversible etiologies of chorea, such as hyperthyroidism, polycythemia vera, or Sydenham’s chorea [14]. Instead of the manifestation of the cause of the chorea itself, it may reflect compensatory changes that intend to inhibit chorea [14]. In our case, chorea responded to risperidone, but first-line agents for symptomatic treatment of Huntington disease-associated chorea and chorea of any cause are inhibitors of presynaptic vesicular monoamine transporter type 2 that cause striatal dopamine depletion [15].In conclusion, we report a peculiar case of hemichorea after ipsilateral frontal cortex infarction. We assume both previous contralateral brain injury and recent impairment of ipsilateral basal ganglia circuit contributed to the strange manifestation. However, the underlying mechanism still needs to be elucidated. More investigations such as functional MRI, diffusion tensor image and motion evoked potentials which are not available in our hospital are recommended in this situation.

## Supplementary Information

**Additional file 1.**

## Data Availability

Not applicable.

## Abbreviations

STN: Subthalamic nucleus
CSF: Cerebrospinal fluid
CBV: Cerebral blood flow
CST: Crossed corticospinal tracts
TTP: Time-to-peak

## References

[1] Chung SJ, Im JH, Lee MC, Kim JS (2004). Hemichorea after stroke: clinical-radiological correlation. J Neurol.

[2] Strauss S, Rafie D, Nimma A, Romero R, Hanna PA (2019). Pure cortical stroke causing hemichorea-hemiballismus. J Stroke Cerebrovasc Dis.

[3] Carbayo Á, Sarto J, Santana D, Compta Y, Urra X (2020). Hemichorea as presentation of acute cortical ischemic stroke. case series and review of the literature. J Stroke Cerebrovasc Dis.

[4] Cotroneo M, Ciacciarelli A, Cosenza D (2020). Hemiballism: unusual clinical manifestation in three patients with frontoparietal infarct. Clin Neurol Neurosurg.

[5] Moersch FP (1939). Hemiballismus. Arch Neurol Psychiatr.

[6] Borgohain R, Singh AK, Thadani R, Anjaneyulu A, Mohandas S (1995). Hemiballismus due to an ipsilateral striatal haemorrhage: an unusual localization. J Neurol Sci.

[7] Kannepalli NR, Yadav R, Vazhayil V, Somanna S, Pal PK (2016). Ipsilateral Hemichorea-hemiballism in a Case of Postoperative Stroke. Tremor Other Hyperkinet Mov (N Y).

[8] Alonso JV, del Pozo FJ, Simon JC, Valenzuela S, Perez Gomez F, Lopera E (2015). Limb-Shaking TIA Presenting as Hemichorea-Hemiballismus: TIA chameleons diagnostic challenge in the emergency department. J Stroke Cerebrovasc Dis.

[9] Hwang KJ, Hong IK, Ahn TB, Yi SH, Lee D, Kim DY (2013). Cortical hemichorea-hemiballism. J Neurol.

[10] Nambu A (2004). A new dynamic model of the cortico-basal ganglia loop. Prog Brain Res.

[11] Inatomi Y, Nakajima M, Yonehara T, Ando Y (2017). Ipsilateral hemiparesis in ischemic stroke patients. Acta Neurol Scand.

[12] Patra DP, Narayan V, Savardekar A, Dossani RH, Cajavilca C, Javalkar V, Gonzalez-Toledo E, Cuellar HH. Acute Supratentorial Ischemic Stroke with Ipsilateral Hemiparesis: Pathomechanism and Management Challenges. World Neurosurg. 2018;119:1-5. 10.1016/j.wneu.2018.07.172. Epub 2018 Jul 30. 10.1016/j.wneu.2018.07.172 30071337

[13] Saada F, Antonios N (2014). Existence of ipsilateral hemiparesis in ischemic and hemorrhagic stroke: two case reports and review of the literature. Eur Neurol.

[14] Ehrlich DJ, Walker RH (2017). Functional neuroimaging and chorea: a systematic review. J Clin Mov Disord.

[15] Bashir H, Jankovic J (2018). Treatment options for chorea. Expert Rev Neurother.

